# Resident physicians’ and Midwives’ Knowledge of Preeclampsia and Eclampsia Reflected in Their Practice at a Clinical Hospital in Southern Romania

**DOI:** 10.25122/jml-2019-0130

**Published:** 2019

**Authors:** Carmen Liliana Soggiu-Duta, Nicolae Suciu

**Affiliations:** 1. Department of Obstetrics-Gynecology, Carol Davila University of Medicine and Pharmacy, Bucharest, Romania; 2. Department of Obstetrics-Gynecology, Alessandrescu Rusescu Institute for Mother and Child Care, Bucharest, Romania

**Keywords:** Resident physicians, Midwives, Knowledge, Preeclampsia, Eclampsia Management, Romania

## Abstract

Romania has drastically improved an array of health indicators in recent years, including maternal mortality rates, which decreased from 1990 to 2015, but the mortality rates are still high, ranking among the first in Europe. Preeclampsia and eclampsia constitute one of the primary causes of maternal death in the country. The study was conducted from early January to the end of February 2019 to assess the current state of resident physicians’ and midwives’ knowledge of preeclampsia and eclampsia reflected in their practice at a clinical hospital in southern Romania. Self-administered questionnaires were used for data collection.

Most of the investigated resident physicians and midwives (87.5%) answered correctly regarding the correct definition of preeclampsia. The first choice of procedure for the patient with preeclampsia (vaginal delivery or C-section) was chosen correctly by only 37.5% of the participants. Regarding the correct identification of the necessary paraclinical tests used for women with suspected preeclampsia, 58.3% of the participants answered correctly. As far as the correct identification of the possible risks of dexamethasone administration to mothers is concerned, only 29.2% of the participants answered correctly. Also, 58.3% of the participants answered correctly regarding the correct identification of contraindicated uterotonic drugs for women with diagnosed hypertension.

Resident physicians and midwives are aware of pregnancy complications, but they hold limited knowledge specific to pregnancy complications as preeclampsia and eclampsia. It is imperative to promote studies to evaluate the impact of enhancing their training to include additional content related to the early detection and management of preeclampsia and eclampsia.

## Introduction

Pregnancy is special, let us make it safe” (World Health Organization, 1998)

### Background of the Study

Maternal mortality is a significant public health issue in Romania, although some of these deaths are related to avoidable conditions. Romania has drastically improved an array of health indicators in recent years, including maternal mortality rates, which decreased from 1990 to 2015, but mortality rates remain still unacceptably high compared to the rest of Europe. Preeclampsia and eclampsia are the first or second cause of maternal and perinatal mortality and morbidity worldwide.

20-25% of maternal deaths are associated with preeclampsia and 10-15% to eclampsia. The incidence of preeclampsia in Europe is 2% [1]. It has an incidence of 1.8% to 16.7% in countries with low and medium incomes [2]. Every day, about 810 women around the world still die of potentially avoidable causes associated with pregnancy and puerperium [3]. It is a challenge to manage the method for early recognition of preeclampsia based on clinical assessment and follow-up examinations during the prenatal visits [4,5]. “In obstetrics, the difference between a careful doctor (or midwife) and a careless one can be very large indeed. The introduction, therefore, of an ordinary standard of good obstetric practice, not necessarily at the level of the hospital specialist, can be expected to have a profoundly beneficial effect in societies that still suffer high maternal mortality” [6].

Hospitals are undergoing constant changes to meet the demands of health care. The most difficult challenge is in developing an organizational infrastructure that supports change, in order to develop quality assurance programs and initiatives for sustainable change, targeted at achieving the best results for patients [7]. The World Health Organization declared in 2009 that some of the examples that need further research to reduce mortality and morbidity are inadequate knowledge, skills, and competences. Healthcare professionals must stay competent because it is necessary to ensure patient safety and warrant care.

Competence is generally defined as knowledge, attitude, and skills. It is, therefore, presented with different integration processes [8]. Competence development is not only essential to acquire knowledge, attitudes and skills, but also to integrate these and acquire professional competences. It is also essential as a prerequisite for the respective work function and successful performance of a professional task. Therefore, knowledge, attitude, and skills should be measured together (e.g., at the same time), since they are evident in actions. To date, there is little international research around the world on knowledge and the regular practices regarding preeclampsia and eclampsia by resident physicians and midwives. The literature available in Romania showed no studies that refer to the resident physicians’ and midwives’ knowledge of preeclampsia and eclampsia reflected in their practice.

The latest international studies show that the limited knowledge of the healthcare professions is directly connected to complications in pregnancy and increases maternal and perinatal mortality [9].

This study aimed to assess the current state of resident physicians’ and midwives’ knowledge of preeclampsia and eclampsia that reflects in their practice at a clinical hospital in southern Romania to identify this critical gap in the literature. This research will help to improve preeclamptic/eclamptic Patients’ care and will result in the reduction of maternal mortality in Romania.

## Material and Methods

### Study design

This is a cross-sectional study that was conducted from early January to the end of February 2019, involving resident physicians and midwives at the department of Obstetrics and Gynecology, “Polizu” Clinical Hospital, Bucharest, Romania.

### Study Sample

The study included 12 resident physicians and 12 midwives. Written informed consent was obtained from all participants and the research was approved by the Ethics Committee of our institution.

### Tools for Data Collection

A two-part questionnaire was used. The first part included demographic data, and the second part was represented by a questionnaire with 31 questions, divided into seven subjects. Each subject is subdivided into further categories developed to evaluate the knowledge of resident physicians and midwives.

### Grading

Thirty-one questions determined the knowledge content of the participants. A score of (1) was assigned for the right answer, and (0) for the wrong one. An answer with (yes) led to (1) and (no) led to (0). The total score of 31 for knowledge has been converted to 100%.

### Analysis of data

The statistical analysis was performed using IBM SPSS Statistics 20 and Microsoft Office Excel/Word 2013.

## Results

### Section A: Description of demographic variables of resident physicians and midwives.

Data from Table 1 shows the demographic values of the investigated resident physicians and midwives. The average age was 35.58 ± 10.97 years, and most of the participants were females (83.3%), everyone having college degrees (100%). The average years of experience of the participants were 12.5 ± 12.535 years.

**Table 1:** Demographic values of the investigated resident physicians and midwives.

**Variable**	
**Age (Average ± SD)**	35.58 ± 10.97 years
**Sex (Nr. / %)**	20 (83.3%) F / 4 (16.7%) M
**Education level (Nr. / %)**	24 (100%) Tertiary
**Years of experience (Average ± SD)**	12.5 ± 12.535 years

### Section B: Knowledge and the regular practices regarding preeclampsia and eclampsia of resident physicians and midwives

**Figure 1:**
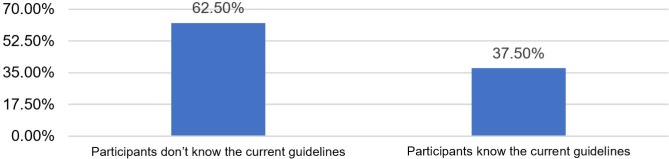
Distribution of the investigated resident physicians and midwives according to the acquaintance of current clinical guidelines for preeclampsia/eclampsia management.

Data from Table 2 and Figure 1 show the distribution of the investigated resident physicians and midwives according to the acquaintance of current clinical guidelines for preeclampsia/eclampsia management. Only 37.5% of the resident physicians and midwives know the current guidelines for preeclampsia/eclampsia management.

**Table 2:** Distribution of the investigated resident physicians and midwives according to the acquaintance of current clinical guidelines for preeclampsia/eclampsia management.

Criteria	Nr.	Percentage
**Participants do not know the current guidelines**	15	62.5%
**Participants know the current guidelines**	9	37.5%

**Figure 2:**
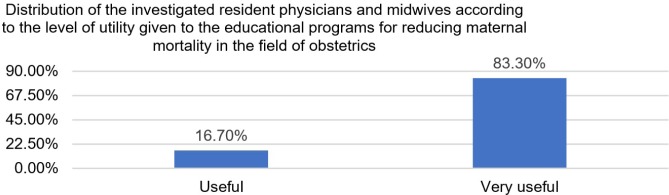
Distribution of the investigated resident physicians and midwives according to the level of utility given to the educational programs for reducing maternal mortality in the field of obstetrics.

Data from Table 3 and Figure 2 show the distribution of the investigated resident physicians and midwives according to the level of utility given to the educational programs for reducing maternal mortality in the field of obstetrics. Most of them consider educational programs very useful (83.3%).

**Table 3:** Distribution of the investigated resident physicians and midwives according to the level of utility given to the educational programs for reducing maternal mortality in the field of obstetrics.

Criteria	Nr.	Percentage
**Useful**	4	16.7%
**Very useful**	20	83.3%

**Figure 3:**
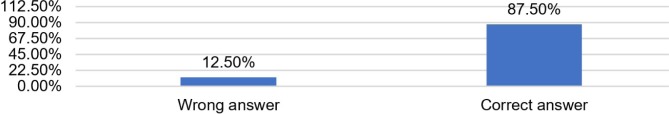
Distribution of the investigated resident physicians and midwives according to the correct identification of the preeclampsia definition.

Data from Table 4 and Figure 3 show the distribution of the investigated resident physicians and midwives according to the correct identification of the preeclampsia definition. Most of them answered correctly (87.5%).

**Table 4:** Distribution of the investigated resident physicians and midwives according to the correct identification of the preeclampsia definition.

Criteria	Nr.	Percentage
**Wrong answer**	3	12.5%
**Correct answer**	21	87.5%

**Figure 4:**
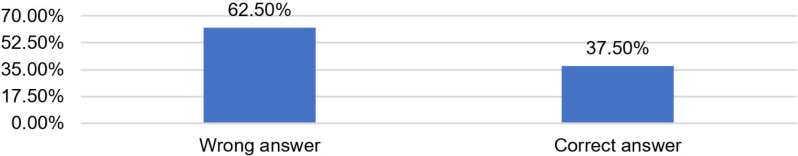
Distribution of the investigated resident physicians and midwives according to the correct identification of the first choice of procedure for the patient with preeclampsia: vaginal delivery or C-section.

Data from Table 5 and Figure 4 show the distribution of the investigated resident physicians and midwives according to the correct identification of the first choice of procedure for the patient with preeclampsia: vaginal delivery or C-section. Only 37.5% of the participants answered correctly.

**Table 5:** Distribution of the investigated resident physicians and midwives according to the correct identification of the first choice of procedure for the patient with preeclampsia: vaginal delivery or C-section

Criteria	Nr.	Percentage
**Wrong answer**	15	62.5%
**Correct answer**	9	37.5%

**Figure 5:**
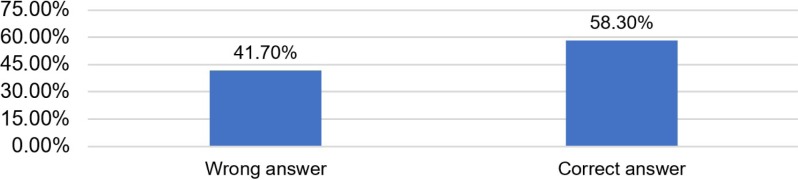
Distribution of the investigated resident physicians and midwives according to the correct identification of the necessary paraclinical tests used for women with suspected preeclampsia.

Data from Table 6 and Figure 5 show the distribution of the investigated resident physicians and midwives according to the correct identification of the necessary paraclinical tests used for women with suspected preeclampsia. 58.3% of the participants answered correctly.

**Table 6:** Distribution of the investigated resident physicians and midwives according to the correct identification of the necessary paraclinical tests used for women with suspected preeclampsia.

Criteria	Nr.	Percentage
**Wrong answer**	10	41.7%
**Correct answer**	14	58.3%

**Figure 6:**
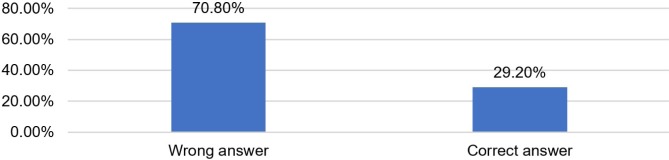
Distribution of the investigated resident physicians and midwives according to the correct identification of the possible risks of dexamethasone administration to mothers.

Data from Table 7 and Figure 6 show the distribution of the investigated resident physicians and midwives according to the correct identification of the possible risks of dexamethasone administration to mothers. Only 29.2% of the participants answered correctly.

**Table 7:** Distribution of the investigated resident physicians and midwives according to the correct identification of the possible risks of dexamethasone administration to mothers.

Criteria	Nr.	Percentage
**Wrong answer**	17	70.8%
**Correct answer**	7	29.2%

**Figure 7:**
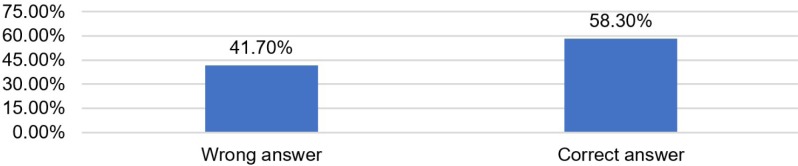
Distribution of the investigated resident physicians and midwives according to the correct identification of contraindicated uterotonic drugs for women with diagnosed hypertension.

Data from Table 8 and Figure 7 show the distribution of the investigated resident physicians and midwives according to the correct identification of contraindicated uterotonic drugs for women with diagnosed hypertension. 58.3% of the participants answered correctly.

Although resident physicians and midwives are aware of pregnancy complications, they hold limited knowledge specific to preeclampsia/eclampsia. There is a need to promote studies to evaluate the impact of enhancing their training to include additional content related to the identification and management of preeclampsia/eclampsia and to reduce the maternal and perinatal mortality in Romania by preeclampsia/eclampsia.

**Table 8:** Distribution of the investigated resident physicians and midwives according to the correct identification of contraindicated uterotonic drugs for women with diagnosed hypertension.

Criteria	Nr.	Percentage
**Wrong answer**	10	41.7%
**Correct answer**	14	58.3%

## Discussion

The average age was 35.58 ± 10.97 years, and most of the participants were females (83.3%), everyone having college degrees (100%), and the average years of experience of the participants was 12.5 ± 12.535 years. Only 37.5% of the resident physicians and midwives know the current guidelines for preeclampsia/eclampsia management, and most of them consider the educational programs very useful for reducing maternal mortality in the field of obstetrics (83.3%).

Most of the investigated resident physicians and midwives (87.5%) answered correctly according to the preeclampsia definition. Regarding the first choice of procedure for the patient with preeclampsia (vaginal delivery or C-section), only 37.5% of the participants answered correctly. 58.3% of the participants answered correctly regarding the necessary paraclinical tests used for women with suspected preeclampsia. Only 29.2% of the participants identified the possible risks of dexamethasone administration to mothers. Concerning the correct identification of contraindicated uterotonic drugs for women with diagnosed hypertension, 58.3% of the participants answered correctly. Such results show inadequate knowledge and awareness of preeclampsia and its related complications, despite being considered one of the leading causes of maternal death [10-13]. This study shows that there is a large gap between resident physicians’ and midwives’ perceptions of preeclampsia and eclampsia and the biomedical perspective.

The fact that the resident physicians and midwives included in the study still have misconceptions regarding preeclampsia/eclampsia reinforces the need for “intensive education of the pregnant women by the health worker, as stated by James et al. [14].

This highlights the need for clear knowledge among resident physicians and midwives to ensure improved education and skills.

This calls for better communication of guidelines, especially in regions where there are increasing initiatives to redistribute responsibilities for health care through the sharing of tasks. A midwife is defined as a person who is ‘qualified and competent to independently practice midwifery in the manner and to the level prescribed and who is capable of assuming responsibility and accountability for such practice’ [15].

It is problematic if midwives functioning independently at a tertiary care center level in Bucharest, the capital and largest city of Romania are not able to distinguish between the various categories of hypertensive disorders of pregnancy, diagnose, assess or manage patients with preeclampsia/eclampsia. In this study, despite the fact that all the participants were fully qualified midwives, the majority had no knowledge about the correct way of managing patients with preeclampsia/eclampsia. Thus, it is critical that midwives have the required knowledge and skills to function independently without the support of a doctor and are able to refer to the next level of care when required. An early diagnosis may improve the outcome of pregnancy as better maternal and fetal monitoring may lead to earlier identification of clinical signs of the disease, and treatment may be given where necessary [16].

## Conclusions

The results of this study illustrate that resident physicians and midwives are not aware of pregnancy complications and have limited knowledge with regards to preeclampsia and eclampsia. The findings of the study highlighted some of the important aspects, such as that none of the resident physicians or midwives reached an excellent level of performance (Score 100% - Level of knowledge).

Researchers need to be supported to assess the impact of improving their preparation to include additional content linked to early detection and management of preeclampsia and eclampsia. Resident physicians and midwives are a very important point to refer to the optimal care of obstetric emergencies. Resident physicians and midwives in this study were not capable of identifying and initiating appropriate care for women with preeclampsia/eclampsia. Such competencies, combined with training and equipment availability, could improve maternal health in Romania. Regular training and retraining are needed to enable effective task-sharing with the resident physicians and midwives.

The study illustrates that knowledge of preeclampsia and eclampsia are limited amongst resident physicians and midwives in Romania; there are gaps in knowledge regarding the management and treatment of important conditions. It also highlights the need for a review of maternal health policies in Romania and the need for health care providers to be equipped with appropriate skills and relevant materials to provide medical education and sensitization to improve maternal and perinatal health.

Further research is required to prepare lesson strategies to assess the learning and commitment of health promoters regarding obstetric emergencies. Simulation training in hospitals helps to identify error sources in daily teamwork to improve safety for the mother and child during emergencies. Clinical guidelines to involve evidence-based practices should be planned well and created explicitly and transparently based on clear foundations. The clinical guidelines for preeclampsia/eclampsia should help avoid complications during pregnancy and improve maternal and fetal outcomes.

## Conflict of Interest

The authors confirm that there are no conflicts of interest.
